# Significant discharge of CO_2_ from hydrothermalism associated with the submarine volcano of El Hierro Island

**DOI:** 10.1038/srep25686

**Published:** 2016-05-09

**Authors:** J. M. Santana-Casiano, E. Fraile-Nuez, M. González-Dávila, E. T. Baker, J. A. Resing, S. L. Walker

**Affiliations:** 1 Instituto de Oceanografía y Cambio Global (IOCAG). Universidad de Las Palmas de Gran Canarias. Las Palmas de Gran Canaria. Spain; 2 Instituto Español de Oceanografía. Centro Oceanográfico de Canarias. 38180 Santa Cruz de Tenerife. Spain; 3 Joint Institute for the Study of the Atmosphere and Ocean, University of Washington, and Pacific Marine Environmental Laboratory, NOAA, Seattle WA, USA; 4 Pacific Marine Environmental Laboratory, NOAA, Seattle WA, USA

## Abstract

The residual hydrothermalism associated with submarine volcanoes, following an eruption event, plays an important role in the supply of CO_2_ to the ocean. The emitted CO_2_ increases the acidity of seawater. The submarine volcano of El Hierro, in its degasification stage, provided an excellent opportunity to study the effect of volcanic CO_2_ on the seawater carbonate system, the global carbon flux, and local ocean acidification. A detailed survey of the volcanic edifice was carried out using seven CTD-pH-ORP tow-yo studies, localizing the redox and acidic changes, which were used to obtain surface maps of anomalies. In order to investigate the temporal variability of the system, two CTD-pH-ORP yo-yo studies were conducted that included discrete sampling for carbonate system parameters. Meridional tow-yos were used to calculate the amount of volcanic CO_2_ added to the water column for each surveyed section. The inputs of CO_2_ along multiple sections combined with measurements of oceanic currents produced an estimated volcanic CO_2_ flux = 6.0 10^5^ ± 1.1 10^5 ^kg d^−1^ which is ~0.1% of global volcanic CO_2_ flux. Finally, the CO_2_ emitted by El Hierro increases the acidity above the volcano by ~20%.

The knowledge and quantification of heat and chemicals fluxes from vent fields requires information about the hydrothermal discharges in these systems. Hydrothermal vents discharge discrete jets of undiluted high-temperature fluids or diffuse seepage of low-temperature diluted fluids^1^. Although most research has been concerned with discrete discharge, some studies have focused on diffuse emissions^2^. Data on the magnitude of diffuse flow and its role in geochemical fluxes to the global ocean are limited^1^,^3^,^4^,^5^,^6^,^7^. However, it has been suggested that the geochemical influence of low-temperature diffuse flow to the global ocean could exceed that of high-temperature flow^8^. Although hydrothermalism is commonly associated with volcanic islands, coastal and shallow hydrothermal systems are still poorly studied compared to deep-water mid-ocean ridge systems. The existence of shallow submarine hydrothermal venting has been revealed by the presence of streams of gas bubbles off volcanic islands^9^ such as White Island, (New Zealand)^10^,^11^, Papua New Guinea^12^, Iceland^13^, Milos, Greece^14^, Panarea, (Italy)^15^, and the Azores^9^.

Studies of hydrothermal activity on submarine volcanoes along volcanic arcs also reveal a great diversity of hydrothermal activity^16^ at sites such as NW Eifuku^17^, NW Rota-1^18^,^19^, and Suiyo Seamount^20^. Investigations have also been carried out on hydrothermal activity associated with intra-plate volcanoes, including the Society Islands^21^, Hawaii^22^, Loihi^23^, and Vailulu’u^24^.

Submarine volcanoes exhibit a wide range of CO_2_ to pH relationships, from the venting of acid-rich fluids, to CO_2_-rich fluids, to fluids rich in alkalinity and CO_2_^19^. The shallow depths of many of these volcanoes result in the emplacement of their volcanic and hydrothermal products into the surface oceans where they influence surface ocean chemistry and productivity. However, in places where gas bubbles or hot plumes are not easily observed, the detection and sampling of emissions, either discrete or diffuse, is required to evaluate the magnitude of a volcano’s contribution to the global budget of gas emissions.

Here we present a study of chemical anomalies associated with hydrothermal emissions in the shallow waters around the submarine volcano of El Hierro. Oxidation-Reduction Potential (ORP) and pH sensors were used in combination with a Conductivity-Temperature-Depth (CTD) package in tow-yo surveys in order to define the area affected by the hydrothermal emissions and to obtain a map of ORP and pH anomalies. In addition, we conducted a temporal study on variations of the physical-chemical properties within the plumes related to physical processes. Together, these data provided a direct constraint on the role played by CO_2_ emissions from the hydrothermal activity associated with volcanic islands and their contribution to ocean acidification. These data comprise the first detailed survey of pH and ORP around the summit of a shallow submarine volcano.

## Regional Setting

**Figure 1:**
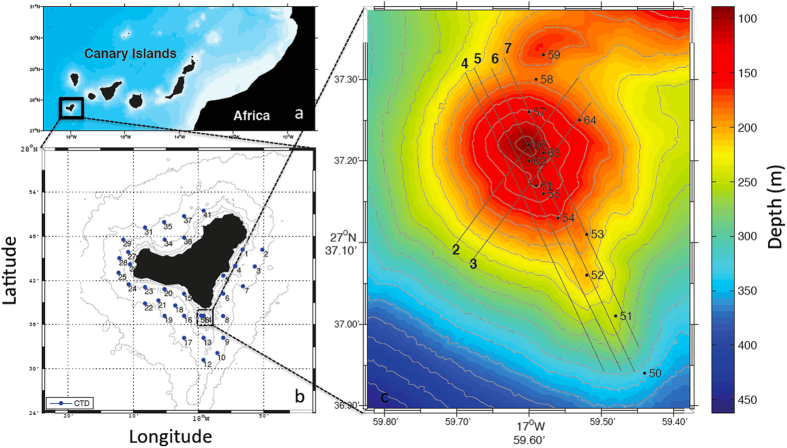
(**a**) Location of El Hierro volcanic edifice within the Canary Archipelago. (**b**) Island of El Hierro and the stations sampled. (**c**) Location of the tow-yos and CTD stations. The maps were generated using Matlab 7.12 R2011a.

The Canary Islands, off northwest Africa (Fig. 1a), were constructed in the Early Miocene as the African plate moved over a mantle hotspot^25^. The islands show a general age progression from the eastern islands (>20 Ma) toward El Hierro (<2 Ma)^26^. The oldest subaerial rocks in El Hierro have been dated at 1.12 ±  0.02 Ma^27^. This island is the emergent summit of a volcanic shield that rises from 3800–4000 m depth and grows up to 1500 m above sea level. The subaerial part (280 km^2^) includes three convergent volcanic rifts (NE-Rift, NW-Rift and S-Rift)^28^. Sedimentary relicts from the 2011 El Hierro eruption demonstrate unequivocally that the youngest pre-island sediment is located beneath El Hierro thereby providing crucial support for an east-west age progression of the onset of volcanism in the Canary archipelago. The fossil evidence from El Hierro therefore concludes the current debate on the origin of the Canary Islands and reinstates a mantle-plume as the most plausible cause of volcanism in the Canary Islands^29^.

The submarine volcano of El Hierro is located 1.8 km south of the Island of El Hierro (Fig. 1a,b). The submarine volcano begins on the S-Ridge at 350 m depth and rises up to 88 m below sea level. It was erupting molten material for five months from October 2011 to March 2012, changing drastically the physical-chemical properties of the water column in the area. After this eruption, the system evolved to hydrothermal vents^30^,^31^.

## Survey of the volcanic edifice

The production of a hydrothermal emission map of the El Hierro edifice required a detailed survey of the area that was carried out through a tow-yo study. From the base to the top of the submarine volcano, an instrument package was towed in a sawtooth pattern by continual lowering and raising between 3 m and 40 m above the seafloor. To delimit the area of anomalies due to hydrothermal emissions, seven SeaBird 911+ CTD-pH-ORP tow-yos were carried out in the area. Four parallel tow-yos followed the topography of the principal and secondary cones and another two tow-yos were perpendicular to these. Tow-yo 6 repeated tow-yo 1 (Fig. 1c). In order to investigate the temporal variability of the system, two CTD-pH-ORP yo-yo studies (raising and lowering the CTD at one location) were carried out from the surface to 3 m above the seabed at two stations (st. 56 and st. 61, Fig. 1c) with 38 and 61 casts, respectively. The time between each cast was 10 minutes and the total duration of the yo-yo study was 6 hours for st. 56 and 10 hours for st. 61. For station 61, three water column sampling casts for discrete pH, total alkalinity (A_T_) and total dissolved inorganic carbon (C_T_) were carried in the maximum anomalies detected with the temperature (T), ORP, and pH sensors. Additionally, 50 stations were occupied around the island of El Hierro outside of the volcano’s influence.

## Use of ORP and pH sensors to detect changes in the redox and acid-base properties of seawater

**Figure 2:**
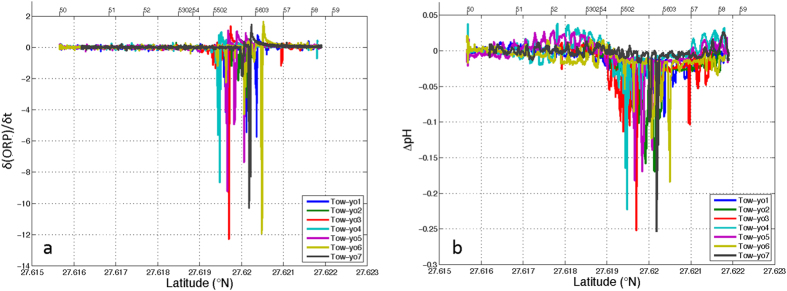
Plots of (**a**) δ (ORP)/δ t and (**b**) Δ pH versus latitude obtained from tow-yos 1–7.

The detection of emissions from low-temperature hydrothermal vents, where the increase in temperature is not evident, requires the utilization of alternative sensors that respond to emissions of certain chemical species. The character of both reduced and acid fluids in the volcanic emissions in the El Hierro submarine volcano^31^ allowed us to detect anomalies related with changes in the chemical potential and the proton concentration using ORP and pH sensors, respectively. Tow-yos with these sensors provided the approximate locations of the emissions as can be seen in the plot of δ (ORP)/δ t and Δ pH versus the latitude (Fig. 2). The ORP sensor responds very quickly to the presence of reduced chemicals in the water column. Changes in potential are proportional to the amount of reduced chemical species present in the water. Here we examine the magnitude of this change by the time derivative of ORP, δ (ORP)/δ t. For pH changes, the mean pH for each depth at a reference station in an area not affected by the vent emission was subtracted from each point measured near the volcanic edifice, defining in this way Δ pH. The signal of the two sensors differs because they respond to different chemical processes. The ORP sensor detects changes in the electrochemical potential due to the emission of reduced species (equation 1) and the pH sensor responds to changes in the H^+^ concentration due to both CO_2_ and acid emissions (equation 2)


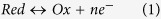



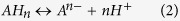


where *Red* refers to reduced species (Fe^+2^, HS^−^ and H_2_), and *AH*_*n*_ refers to acidic compounds (HCl, CO_2_, etc).

## Results

### Delimiting the seawater volume affected by hydrothermal emissions

Anomalies in pH and ORP measurements followed the same pattern in latitude (Fig. 2) and longitude, which indicates that the emissions of both acid and reduced components must be produced in the same area. Although this study was carried out two years after the eruption of molten material had ceased and a degasification phase had begun^31^, the negative pH anomalies observed in the area, including values as high as −0.25, cannot be explained by the arrival of water from the surrounding depths. The pH value of 7.89 in the volcano area is found in the non-affected surrounding water at a depth of 900 m. Moreover, the ORP sensor detected changes that can only be explained by the presence of reduced species due to the existence of vents because this is an area of open waters and its potential must correspond to that of an oxic environment. Hydrothermal ORP anomalies are generally restricted to within ~1 km of their source because of the rapid oxidation of the reduced species^32^.

Anomalies of both pH and ORP obtained during the 7 tow-yos over the volcano provided a contour map of the affected surface. In order to quantify this surface, two longitudinal (2–3) and four meridional (4–7) tow-yo sections were used. Using a Mat lab routine and considering that the pH anomalies should be higher than − 0.04, the affected area was 13,000 ±  800 m^2^. The volume, 206,000 ±  50,000 m^3^, was calculated considering the real contoured affected area in each tow-yo transect (i.e., between 3 and 40 m above bottom).

**Figure 3:**
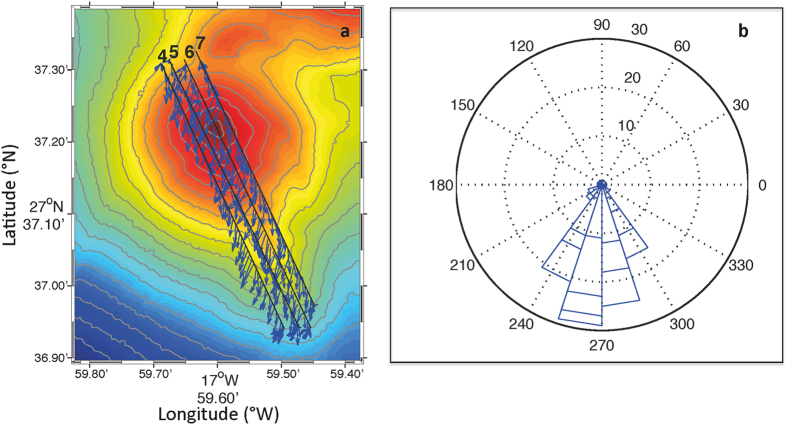
(**a**) ADCP mean average (cm/s) and (**b**) ADCP mean direction for all meridional tow-yo transects carried out on 21st March 2014.

Using a shipboard Acoustic Doppler Current Profiler (ADCP) during the tow-yos over the volcanic edifice, mean velocities, perpendicular to each transect, were calculated for each tow-yo (0.22, 0.17, 0.22 and 0.21 cm s^−1^, respectively for tow-yos 4–7), with a mean velocity =  0.20 ±  0.12 cm s^−1^. Figure 3 shows the current speed and direction for tow-yos 4–7. The current direction was to the south-southwest and was relatively stable for all tow-yos. The tide was not eliminated from the data in order to show that its effect, compared with the predominant direction and intensity of the main current, does not change the direction of the resultant velocity.

### Estimation of the CO_2_ flux from the volcanic edifice

**Figure 4:**
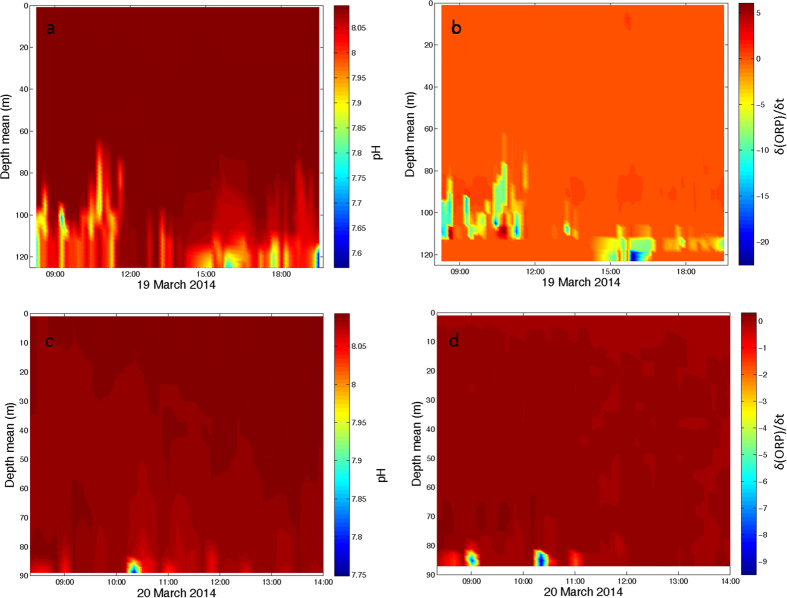
Yo-yo studies carried out at station 61 for (**a**) pH and (**b**) δ (ORP)/δ t and station 56 for (**c**) pH and (**d**) δ (ORP)/δ t.

**Figure 5:**
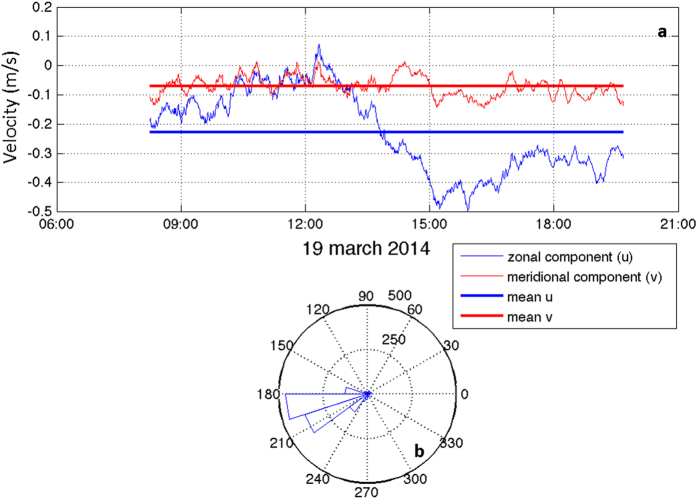
(**a**) ADCP meridional and zonal components of the velocity and (**b**) ADCP mean direction for the yo-yo 61 station.

Repetitive yo-yos at stations 56 and 61 confirm significant emissions of CO_2_ from the volcano and indicate a significant fluctuation in the carbon system variables (in pH and in both C_T_ and A_T_ when measured) associated mostly with changes in local currents. The yo-yo studies carried out at station 61 (Fig. 4a,b) and 56 (Fig. 4c,d) showed that changes in the current intensity and direction produced a discontinuous signal in pH and δ (ORP)/δ t. St. 56 is located at the top of the cone and st. 61 on one of the flanks. During the yo-yo studies, higher anomalies were observed at st. 61 than at st. 56. Figure 5 shows both the intensity and direction of the current for yo-yo 61. For the first 6–8 hrs of yo-yo sampling little variation in current direction or velocity was observed. The tidal mode at this site is M2, with a period of 12 hours and negligible velocity relative to the predominant local mean current^33^. However, an increase in the velocity of the zonal component in the second half of the record could explain the pattern of the anomalies observed in Fig. 4a,b. When current velocity was low, as during the first hours of the study, the anomalies were greater than when the current velocity intensity increased.

**Figure 6:**
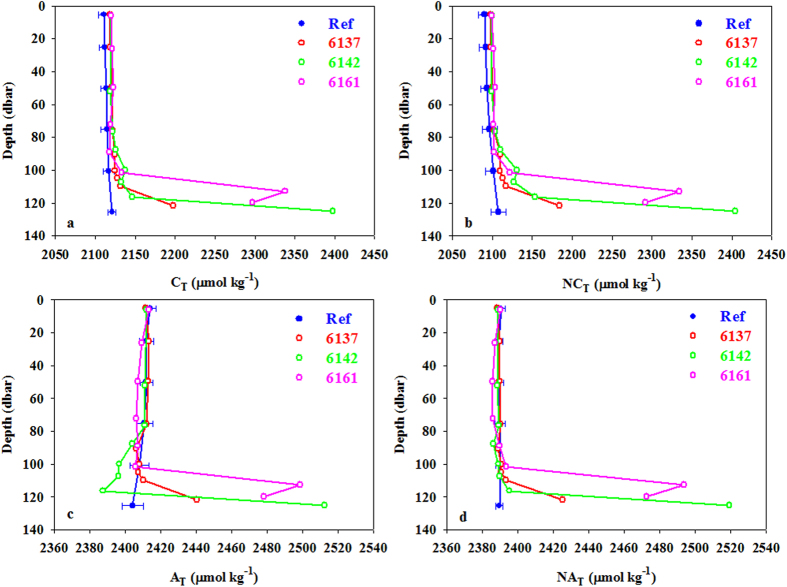
Carbonate variables, (**a**) C_T_, (**b**) NC_T_, (**c**) A_T_ and (**d**) NA_T_, expressed in μ mol kg^−1^ at station 61, taken at three different yo-yos (37, 42 and 61) for the yo-yo series. NC_T_ and NA_T_, were normalized at a constant salinity of 36.5.

At st. 61, discrete samples were taken at three different times when the sensors registered the highest anomalies in order to measure pH, alkalinity, and total dissolved inorganic carbon. For these three variables, anomalous values were found close to the bottom. pH values were lower than those expected at this depth, and C_T_ and A_T_ values higher than usual. C_T_ and A_T_ were then normalized to a constant salinity (NX_T _=  (X_T_/S)*36.5) in order to avoid the salinity effect due to the possible advection of different water masses. The normalized values showed the anomalies were not removed (Fig. 6). On yo-yo 6142, the NC_T_ increased from 2100 μ mol kg^−1^ to 2400 μ mol kg^−1^ and the NA_T_ from 2390 μ mol kg^−1^ to 2520 μ mol kg^−1^, at a depth of 120 m, close to the bottom, indicating that the hydrothermal fluid producing these changes was ~57% CO_2_ and 43% carbonate alkalinity (HCO_3_^−^, CO_3_^2−^). The same was observed for yo-yos 6137 at 120 m depth and 6161 between 110 and 120 m depth, but to a lesser degree. The decreases in pH of 0.08, 0.25, and 0.34 units for three yo-yos (Table 1) were therefore the result of the injection of a fluid rich in both carbonate alkalinity and CO_2_ gas. This is further supported by the linear relationship between Δ C_T_ and Δ pH obtained from the data in Table 1 following Resing *et al.*^19^, which has a slope of − 842 ±  35 (r^2^ =  0.982). If only CO_2_ is added, the slope of Δ C_T_ versus Δ pH is approximately 334 μ mol kg^−1^/pH unit (Supplementary Fig. S1). The slope of − 842 arises from the addition of a hydrothermal source fluid rich in CO_2_ and carbonate alkalinity in a ~60–40% ratio. Figure 6 shows that the emissions of this hydrothermal area acted as an irregular source of CO_2_ (Table 1). The corresponding changes in pH varied from 0.082 to 0.344 due to the increase in the proton concentration. The total proton concentration increased between 20% (cast 6137) and 120% (cast 6142), strongly contributing to acidifying the system.

Distribution maps of the surface affected by the emissions can be obtained from the anomalies of ORP, pH, or T observed in the tow-yo sections. In order to compute the equivalent anomalies for C_T_ over the seven tow-yo sections measured with the sensors the next procedure was followed. Firstly, an equation for the C_T_ reference (C_T_ reference =  2109.66 +  0.042x +  5.35 10^−4^ x^2^–7.81 10^−7^ x^3^, r^2^ =  0.994, where x = depth) was obtained using the measured discrete C_T_ data from 38 stations over the upper 500 m around the El Hierro Island (Fig. 1b). Secondly, a linear relationship between Δ pH (pH *in situ* volcano− pH *in situ* reference) and Δ C_T_ (C_T_ volcano− C_T_ reference) was determined (Δ C_T_ =  − 893.4 Δ pH, r^2^ =  0.999) using all the discrete samples of station 61, including the highest, lowest and non-anomalous values. Through this linear relationship the Δ pH calculated using the pH sensor for the tow-yo sections was transformed to the equivalent Δ C_T_.

**Figure 7:**
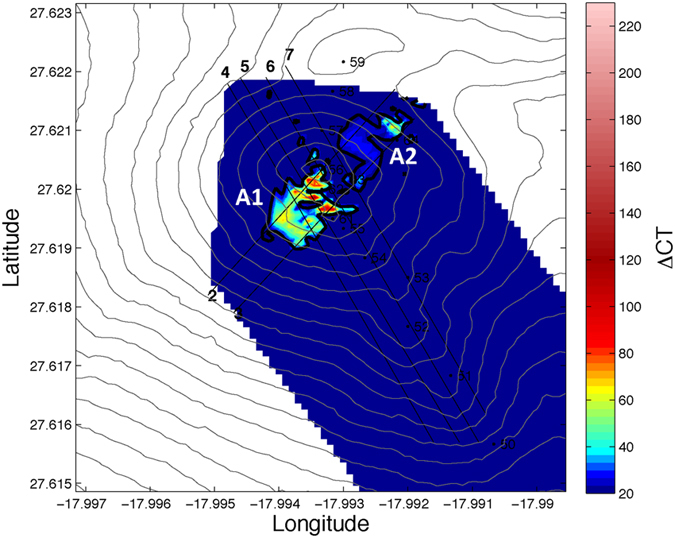
Distribution map of ΔC_T_ showing areas presumably affected by diffuse venting. Principal and secondary affected areas are labelled as A1 and A2, respectively. The map was generated using Matlab 7.12 R2011a.

From a surface map of Δ C_T_ (Fig. 7) it seems that the hydrothermal discharge occurs from a multitude of vents dispersed around the main cone, mainly in two areas (A1 and A2). In these areas, we suggest that the flow is dominated by percolation of vent fluids through the new, highly permeable volcanic edifice. The scale of the anomalies was highly variable near the main cone and the anomaly found in the position of st. 61 during the tow-yo study was not the highest but rather an intermediate anomaly compared with values determined during the yo-yo. The integrated carbon along each tow-yo section was computed and plotted. (Supplementary Fig. S2 shows the anomalies of C_T_ meridional contours and Supplementary Fig. S3 shows the anomalies of C_T_ in the zonal contours.)

Using contours from tow-yos 4–7 (Supplementary Fig. S2) the fluxes of the anomalies in C_T_ were calculated for each tow-yo using the observed anomaly area for every contour, the average anomalies of C_T_, and the current speed normal to the tow-yos for each tow-yo. The average flux of CO_2_ for these four tow-yos is 6.0 10^5^ ±  1.1 10^5^ kg d^−1^ for the whole volume. The same calculation can be carried out for each of tow-yos 4–7 to estimate the variations in the proton concentration. We estimate that the excess CO_2_ in all the affected volume of 206,000 ±  50,000 m^3^ around the volcano produced an integrated 20% increase in seawater acidity.

## Summary and Discussion

The tow-yo studies carried out around El Hierro submarine volcano showed that the anomalies in ORP and pH followed the same pattern in latitude and longitude. Surface maps of anomalies can be defined from these properties and also from the calculated C_T_. The anomalies are all concentrated in a surface area of 13000 ±  800 m^2^ around the main cone of the volcano (area A1 in Fig. 7). This indicates that anomalies of both acid and reduced components must be produced by the hydrothermal emissions of the volcano and not by upwelling water from the surrounding depths. Measurements of the local current velocities support this conclusion. Moreover, from the yo-yo studies, variability in the strength of the emission anomalies is explained by a change in the velocity of the current.

The acidic emissions from the volcanic edifice produced a CO_2_ flux of 6.0 10^5^ ±  1.1 10^5^ kg d^−1^, primarily transported to the southwest. This flux is comparable to that determined from NW Rota-1 (~1 ±  0.25 10^6 ^kg d^−1^), an erupting submarine volcano in the Mariana arc^34^. The global volcanic flux of CO_2_ is estimated^35^ to be ~ 7.2 10^8 ^kg d^−1^ considering mid-ocean ridges, arcs and plumes. These emissions also alter the balance of protons in the surrounding ocean, decreasing pH by ~ 0.25–0.30 units and increasing the seawater acidity by ~20%.

Emissions of CO_2_ gas and carbonate alkalinity from El Hierro accounted for 60% and 40%, respectively, of the pH change in local seawater. When both CO_2_ and carbonate alkalinity are added, pH decreases, but the decrease is less than when only CO_2_ is added. These results are in agreement with previous studies in which carbonate alkalinity from diffuse low-temperature venting accounted for 20% to 40% of the changes observed in pH at submarine volcanoes Ruby, Maug, and Esmeralda^19^.

Sites like these provide an excellent opportunity to study the carbonate system, the volcanic contribution to the global volcanic carbon flux, and the potential environmental impact of these emissions on the surrounding ocean. The flux of CO_2_ from El Hierro submarine volcano (6.0 10^5^ ±  1.1 10^5^ kg d^−1)^) is comparable to that at other submarine volcanoes^34^ but relatively small compared to both the global volcanic flux of CO_2_^35^ (~0.1%) and the anthropogenic flux^36^ (~0.002%). Although CO_2_ flux data from hydrothermally and volcanically active submarine volcanoes in the ocean are scarce, it is estimated that mid-ocean ridge, arcs and plumes contribute^35^ with 2.7 10^8^ kg d^−1^, 3.0 10^8^ kg d^−1^ and 3.6 10^8^ kg d^−1^, respectively. Each of them provides 0.1 ±  0.02 Gt of CO_2_ yr^−1^ to the total CO_2_ added to the ocean. More importantly, however, the emission of CO_2_ from submarine volcanoes generates local ocean acidification that could affect biological communities, with especially important consequences for organisms that use calcium carbonate in their structures.

## Methods

Hydrographic stations were carried out from surface to 1200 m depth with a SeaBird 911+ CTD (Conductivity-Temperature-Depth) with redundant sensors for temperature and conductivity together with light transmission (Chelsea/Seatech), dissolved oxygen (SBE 43), pH (SBE 18), ORP (Oxidation-Reduction-Potential, PMEL-NOAA), pressure, and altimeter sensors. The accuracy of the temperature and conductivity sensors are 0.001 °C and 0.0003 S/m, respectively. Water samples with a 24–12L bottle carousel were carried out at 52 stations for the measurements of O_2_, pH, A_T_ and C_T_. A high-resolution study over the submarine volcano including hydrographic station 50–59 and 61–64 was done. Moreover, 3 yo-yos (over stations 54, 56 and 61) and 7 tow-yos using CTD including the pH and ORP sensors were conducted over the volcanic edifice. Yo-yos and tow-yos are effective methods for mapping and sampling hydrothermal plumes. Both operations consisted in lowering the CTD package to near the bottom, then with the ship in the same position for the yo-yo and with the ship moving for the tow-yo, cycling the package up and down only through the bottom few hundred meters. R/V Ángeles Alvariño, from the Spanish Institute of Oceanography, used a Dynamic Positioning System to conduct tow-yos at 0.2–0.4 kn.

### ADCP

Continuous current measurements were made in the upper waters using a 150 kHz Ocean Surveyor Shipboard Acoustic Doppler Current Profiler, SADCP. The ADCP provides estimates of the horizontal velocity components as a function of depth, based on the Doppler effect of sound wave reflections against small particles carried by ocean currents. Such velocity profiles are obtained approximately every second and averaged over 5 minutes and binned vertically in 8 m increments.

### pH sensor

The SBE 18 pH sensor uses a pressure-balanced glass-electrode/Ag/AgCl-reference pH probe to provide *in-situ* measurements at depths up to 1200 meters. The sensor was initially calibrated against buffer solutions (4, 7, and 10 pH ±  0.02 pH) following the Sea-Bird protocol. In order to express the measurements in total pH scale, all the data were corrected. For this, discrete values of pH, at fixed depth, from the sensor (pH_NBS *in situ*_) were compared with the pH of samples measured in the water column using the m-cresol purple methods for UV-vis spectrophotometric pH (pH_T *in situ*_) obtaining a dependence equation pH_T *in situ*_ =  0.9013 (pH_NBS *in situ*_) +  0.6905 (r^2^ =  0.998).

### ORP sensor

The PMEL ORP sensor has a platinum working electrode and an Ag/AgCl reference electrode located in a single PEEK thermoplastic body. ORP sensor range is − 500 to + 500 millivolts, with output scaled to 0–5 volts for an auxiliary analog channel on the CTD. PMEL ORP sensor does not provide an Eh measurement, however, the sensors respond instantaneously to the presence of reduced chemical species with a decrease in the measured electric potential^37^. ORP data are expressed as a time derivative (δ (orp)/δ t), and anomalies are identified by negative values. This sensor provides a relative measure and no calibration is needed.

### pH_T,25_

The pH was measured on the total scale at a constant temperature of 25 °C (pH_T,25_) by the UV-Vis spectrophotometric technique^38^ that used m-cresol purple as an indicator^39^. The standard deviation for the measurements was ±  0.002.

### Total dissolved inorganic carbon and total alkalinity

The total dissolved inorganic carbon (C_T_) and total alkalinity (A_T_) were determined with a VINDTA 3C system (Marianda, Germany) that used coulometry in order to determine the total dissolved inorganic carbon and differential potentiometry for the total alkalinity^40^,^41^. The certified reference material for oceanic CO_2_, CRMs batch #117 was used to test the performance of both the total inorganic carbon and total alkalinity, resulting in a precision of ± 1.0 μ mol kg^−1^ for both parameters.

## Additional Information

**How to cite this article**: Santana-Casiano, J. M. *et al.* Significant discharge of CO_2_ from hydrothermalism associated with the submarine volcano of El Hierro Island. *Sci. Rep.* **6**, 25686; doi: 10.1038/srep25686 (2016).

## Supplementary Material

Supplementary Information

## Figures and Tables

**Table 1:** Differences with respect to the reference values (regional backgrounds) (Ref) for C_T_, A_T_, pH and *f*CO_2_ for cast 37, 42 and 61 expressed as ΔC_T_, ΔA_T_, ΔpH and Δ*f* CO_2_, respectively.

Station-cast	6137	6142	6161	6161
Depth (m)	121	125	113	120
T (°C)	18.3838	16.8415	17.8663	17.8615
S	36.7198	36.3735	36.5994	36.5529
Ref C_T_	2120.5	2121	2119.1	2120.2
Δ C_T_	77.3	276.8	219.2	176.7
Ref A_T_	2404.6	2404	2406	2404.9
Δ A_T_	35.7	108.3	92.6	73.1
Ref pH	8.078	8.077	8.081	8.079
Δ pH	– 0.082	– 0.344	– 0.25	– 0.201
Ref *f*CO_2_	371.6	372.5	369.1	371.1
Δ *f*CO_2_	101.3	595.2	382.6	288.4

T and S are the values for each station at each depth.
