# Supplementary material for: Significant discharge of CO2 from hydrothermalism associated with the submarine volcano of El Hierro Island
Source: Sci Rep. 2016 May 9;6:25686. doi: 10.1038/srep25686 (PMC4860579; doi:10.1038/srep25686)
Supplement: Supplementary Information [file srep25686-s1.doc]

**Supplementary information**

**Significant discharge of CO_2_ from hydrothermalism associated with the submarine volcano of El Hierro Island**

J. M. Santana-Casiano^1^, E. Fraile-Nuez^2^, M. González-Dávila^1^, E. T. Baker^3^, J. A. Resing^3^ and S. L. Walker^4^

^1^Instituto de Oceanografía y Cambio Global (IOCAG). Universidad de Las Palmas de Gran Canarias. Las Palmas de Gran Canaria. Spain

^2^Instituto Español de Oceanografía. Centro Oceanográfico de Canarias. 38180 Santa Cruz de Tenerife. Spain

^3^Joint Institution for the Study of the Atmosphere and Ocean, University of Washington, and Pacific Marine Environmental Laboratory, NOAA, Seattle WA, USA

^4^Pacific Marine Environmental Laboratory, NOAA, Seattle WA, USA


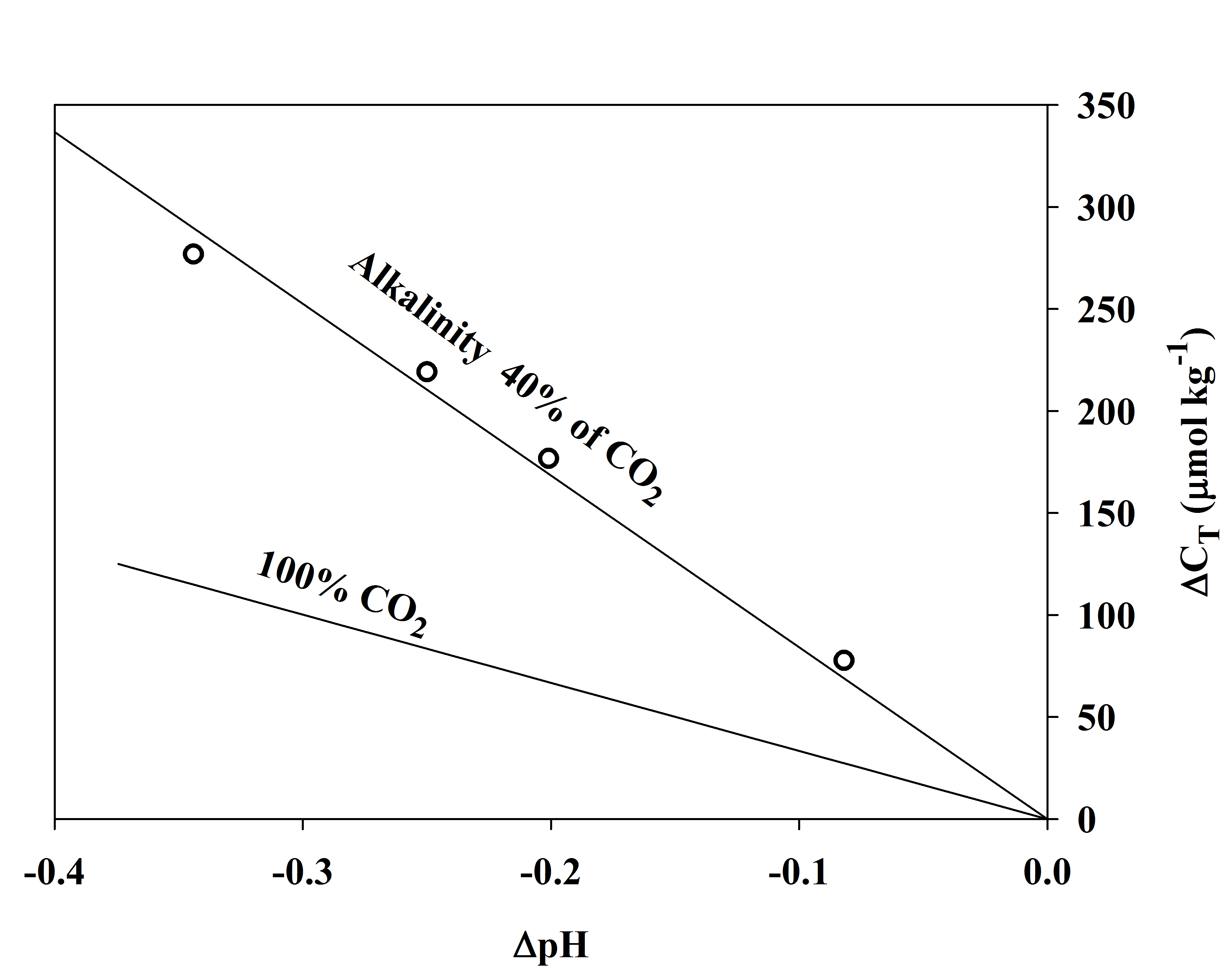


Figure S1. Lineal relationship between ΔC_T_ and ΔpH obtained from the data in Table 1 following Resing et al., 2009. When only CO_2_ is added, the slope of ΔC_T_ versus ΔpH is 334 μmol kg^-1^/pH unit.


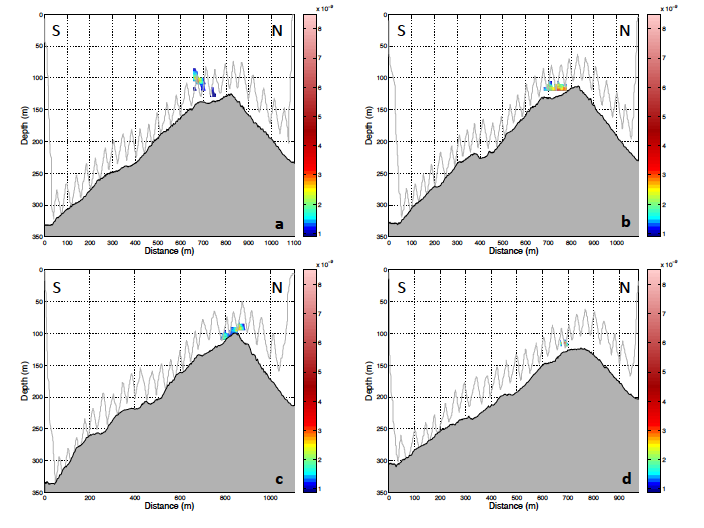


Figure S2. Meridional contours of ΔC_T_ for the four meridional tow-yo transects (4-7) over the submarine volcano.


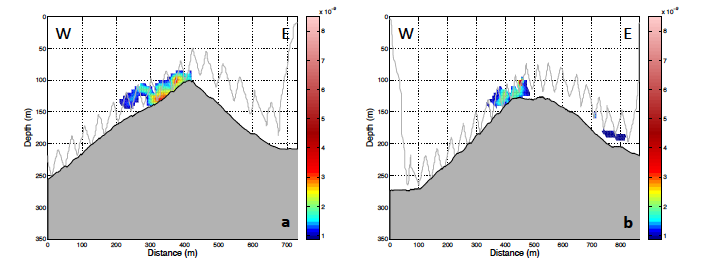


Figure S3. Zonal contours of ΔC_T_ for the two zonal tow-yo transects (2-3) over the submarine volcano.
